# Synthesis and Cytotoxicity Evaluation of 13-*n*-Alkyl Berberine and Palmatine Analogues as Anticancer Agents

**DOI:** 10.3390/molecules171011294

**Published:** 2012-09-25

**Authors:** Lei Zhang, Jingjing Li, Fei Ma, Shining Yao, Naisan Li, Jing Wang, Yongbin Wang, Xiuzhen Wang, Qizheng Yao

**Affiliations:** 1 School of Pharmacy, China Pharmaceutical University, Nanjing 210009, Jiangsu, China; Email: zhanglei04402@163.com (L.Z.); leejingjing119@yahoo.com.cn (J.L.); linaisan@vip.sina.com (N.L.); wangjing0642320@126.com (J.W.); wybhzsyq@126.com (Y.W.); 2 Oil Crops Research Institute, Chinese Academy of Agricultural Sciences, Wuhan 430062, Hubei, China; Email: mafeicpu@163.com; 3 Key Laboratory of Biology and Genetic Improvement of Oil Crops, Ministry of Agriculture, Wuhan 430062, Hubei, China; 4 Wuxi JC Pharmaceutical Technology Inc., Wuxi 214036, Jiangsu, China; Email: yao_sn@yahoo.com.cn; 5 School of Pharmacy, Nanjing Medical University, Nanjing 210029, Jiangsu, China; Email: showshow828@163.com

**Keywords:** berberine, palmatine, alkylation, cytotoxicity, antitumor

## Abstract

By introducing long carbon-chain alkyl groups at the C-13 position of berberine and palmatine, 13-*n*-hexyl/13-*n*-octyl berberine and palmatine chloride analogues **4a**–**d **were synthesized and examined by MTT assays for cytotoxic activity in seven human cancer cell lines (7701QGY, SMMC7721, HepG2, CEM, CEM/VCR, KIII, Lewis), yielding IC_50_ values of 0.02 ± 0.01–13.58 ± 2.84 μM. 13-*n*-Octyl palmatine (compound **4d**) gave the most potent inhibitor activity, with an IC_50_ of 0.02 ± 0.01 μM for SMMC7721. In all cases, the 13-*n*-alkyl berberine and palmatine analogues **4a**–**d** were more cytotoxic than berberine and palmatine. In addition, compounds **4a**–**d **also exhibited more potent cytotoxicity than berberine and palmatine in mice with S180 sarcoma xenografted *in vivo*. The primary screening results indicated that the 13-*n*-hexyl/13-*n*-octyl berberine and palmatine analogues might be valuable source for new potent anticancer drug candidates.

## 1. Introduction

**Figure 1:**
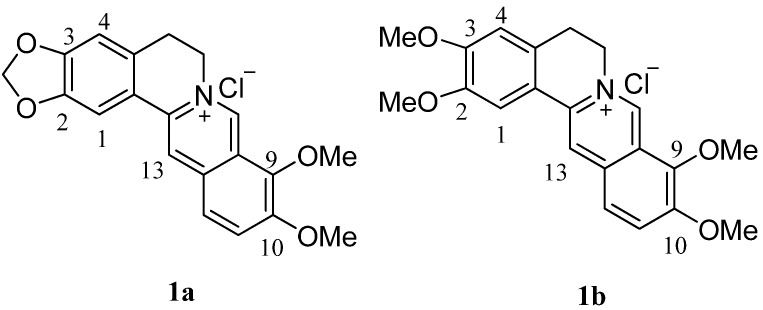
Structures of the lead compounds **1a** and **1b**.

Berberine (**1a**, Figure 1), an isoquinoline alkaloid isolated from the roots and stem bark of *Berberis* species, is widely used as a traditional medicine for treating diarrhea [1] and gastrointestinal disorders [2].

Berberine possesses various biological activities like antibacterial [3,4], antifungal [5], antimalarial [6], antileishmanial [7], anticancer [8,9,10,11,12], anti-Alzheimer’s disease [13,14], antiviral [15,16], cholesterol lowering effect [17] and hypoglycemic effect [18]. Different activities of a number of 8-, 9- and 13-substituted analogues of berberine have been reported. Various analogs using different chain lengths and terminal amino groups have been synthesized to study the DNA-binding afﬁnity or as G-quadruplex stabilizing ligands [19,20,21,22,23,24,25,26].

In addition, palmatine (**1b**, Figure 1) is a close structural analog of berberine. The alkaloid has been used in the treatment of jaundice, dysentery, hypertension, inﬂammation, and liver-related diseases [27]. It has been reported that like berberine, some of the 8- and 13-alkyl analogues of palmatine also possess antimicrobial and antimalarial activities [28]. Palmatine also has significant antitumor activity against HL-60 leukemic cells [29].

In this study, berberine and palmatine analogues were first designed and synthesized by means of the introduction of long carbon-chain alkyl at carbon atom C-13 to improve the antiproliferative activity *in vitro* and *in vivo*.

## 2. Results and Discussion

### 2.1. Chemistry

**Scheme 1:**
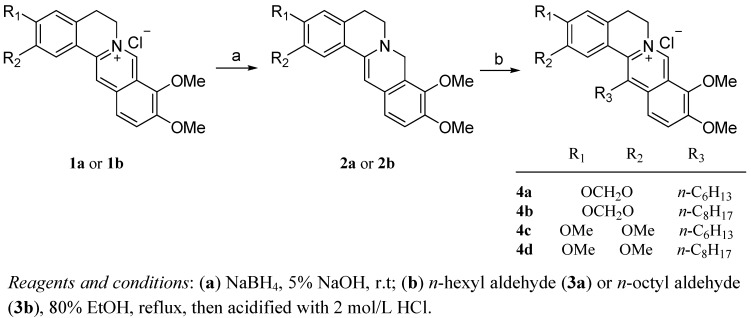
Synthesis of the 13-*n*-alkyl substituted berberine and palmatine analogues **4a**–**d**.

The synthesis of the 13-*n*-alkyl substituted berberine and palmatine analogues involved two steps (Scheme 1). Treatment of commercially available berberine (**1a**) or palmatine (**1b**) with NaBH_4_in 5% NaOH at room temperature gave the key reduced intermediates **2a**,**b**, the dihydrogenated products of **1a** and **1b [30]**. Then, compounds **2a**,**b** were reacted with *n*-hexyl aldehyde (**3a**) and *n*-octyl aldehyde (**3b**), respectively, in a EtOH and HOAc solvent mixture, and then acidiﬁed with 2 mol/L HCl to yield the desired compounds **4a**–**d**, which were fully characterized by ^1^H-NMR, LR-MS and elemental analysis. 

### 2.2. Biology

#### 2.2.1. *In Vitro* Cytotoxic Effects

The cytotoxicity of berberine and palmatine and the herein presented analogues **4a**–**d** were compared by means of a colorimetric microculture assay (MTT assay) in seven human cancer cell lines: 7701QGY, SMMC7721, HepG2 (human hepatoma), CEM (human acute lymphoblastic leukemia), CEM/VCR (vincristine-resistant CEM), K III (mice melanoma) and Lewis (mice lung carcinoma), yielding IC_50_ values mostly in the low micromolar or even submicromolar range (Table 1).

**Table 1:** Cytotoxicity of berberine and palmatine analogues **4a**–**d** compared with berberine and palmatine in various cancer cell lines.

Comp.	IC_50_ (μM) ^a^						
	7701QGY	SMMC7721	HepG2	CEM	CEM/VCR	K III	Lewis
**4a**	3.28 ± 0.27	0.37 ± 0.07	4.74 ± 0.48	3.64 ± 0.35	9.65 ± 2.37	25.47 ± 2.95	2.26 ± 0.49
**4b**	1.79 ± 0.26	0.04 ± 0.02	3.16 ± 0.39	0.37 ± 0.03	5.19 ± 0.64	13.58 ± 2.84	0.86 ± 0.10
**4c**	10.09 ± 1.98	0.68 ± 0.05	5.54 ± 0.24	1.97 ± 0.09	17.54 ± 1.98	30.58 ± 1.69	2.86 ± 0.54
**4d**	1.08 ± 0.25	0.02 ± 0.01	2.28 ± 0.37	0.16 ± 0.11	4.80 ± 0.81	10.41 ± 2.58	0.34 ± 0.09
**berberine**	22.18 ± 1.12	2.09 ± 0.25	117.63 ± 3.13	45.04 ± 1.42	120.37 ± 3.84	84.29 ± 3.42	20.29 ± 4.42
**palmatine**	ND	23.19 ± 1.21	ND	5.68 ± 0.27	230.76 ± 5.21	74.13 ± 4.50	30.18 ± 2.76

ND = not determined. ^a^ 50% inhibitory concentrations in the MTT assay (72 h exposure). Values are means standard deviations obtained from at least two (mostly three) independent experiments.

13-*n-*Hexyl/13-*n*-octyl berberine and palmatine analogues **4a**–**d** exhibited more potent cytotoxicity than berberine and palmatine in all seven cell lines. 13-*n*-octyl-berberine (**4b**) and 13-*n*-octyl-palmatine (**4d**) exerted the most potent antitumor activities, with IC_50_ values of 0.02 ± 0.01–13.58 ± 2.84 *μ*M against various cancer cell lines, and 6-fold stronger antitumor activities than berberine and palmatine. Notably, compound **4d** gave the most potent inhibitor activity, with an IC_50_ of 0.02 ± 0.01 *μ*M for SMMC7721. Compared with the lead compounds, berberine analogues **4a**,**b** showed similar activity to palmatine analogues **4c**,**d**, which exhibited more potent activity against CEM and CEM/VCR cells (vincristine-resistant) lines. These results indicate that the alkylation of the natural products, berberine and palmatine, such as introduction of longer carbon-chain alkyl groups at the C-13-position, can remarkably enhance the antitumor activity. This modification is likely to make the compounds more lipophilic, which may increase the permeability of the cell membrane and improve the bioavailability of the lead compounds. Very recently, the new insights into 13-position substitution of berberine in enhancing the DNA binding had been reported by Kumar and colleagues [26], suggesting that 13-*n*-hexyl/13-*n*-octyl berberine and palmatine analogues may enhance the DNA binding action. The further investigation whether the long carbon-chain substituted berberines and palmatines could enhance the DNA binding action to generate more potent antitumor activity will be reported in due course.

#### 2.2.2. *In Vivo* Anticancer Activity

Anticancer activity *in vivo* was investigated in the murine sarcoma S180 xenografted model on male Kunming mice using intraperitoneal (ip) injection using berberine, palmatine and cyclophosphamide (CP) as the positive control (Table 2). 

**Table 2:** Tumor inhibitory rate of berberine and palmatine and their analogues **4a**–**d** in the murine sarcoma S180 xenografted model.

Comp.	Dose mg/kg	Injection	Number of mice		Weight of mice (g)		Weight of tumor *X*±SD (g)	Tumor inhibitory rate (%)
			Start	End	Start	End		
**Control**	-	iv	10	10	19.48 ± 1.45	22.19 ± 2.20	2.20 ± 0.93	-
**CP**	30	iv	10	10	20.23 ± 1.25	20.81 ± 2.31	0.43 ± 0.28 **	80.61
**berberine**	30	ip	10	9	20.20 ± 1.26	17.66 ± 3.24	1.26 ± 0.54 **	42.99
**4a**	1	ip	10	9	19.98 ± 1.33	17.86 ± 2.25	1.32 ± 0.63 **	40.00
	2.5	ip	10	6	19.62 ± 1.52	18.04 ± 2.54 *	1.01 ± 0.48 **	54.09
**4b**	1	ip	10	8	20.20 ± 2.22	19.17 ± 1.12	1.02 ± 0.25 **	53.52
	2.5	ip	10	5	19.89 ± 1.54	17.11 ± 1.06	0.88 ± 0.28 **	59.86
**palmatine**	30	ip	10	10	20.56 ± 2.03	20.95 ± 2.18	1.45 ± 0.31 **	34.09
**4c**	5	ip	10	10	20.33 ± 1.28	20.45 ± 1.45	1.43 ± 0.44 **	34.88
	10	ip	10	9	19.16 ± 1.21	18.88 ± 2.60 *	1.16 ± 0.44 **	47.40
**4d**	5	ip	10	9	20.72 ± 1.45	19.15 ± 2.75	1.28 ± 0.38 **	42.05
	10	ip	10	5	20.04 ± 1.57	18.63 ± 3.20	1.08 ± 0.42 **	50.96

SD: standard deviation; * *p *< 0.05 *vs.* model group; ** *p *< 0.01 *vs.* model group.

As listed in Table 2, most of the 13-*n*-hexyl/13-*n*-octyl berberines and palmatines showed stronger antitumor activity and higher toxicity than their parent compounds, respectively. The 13-*n*-octyl isoquinoline alkaloid derivatives **4b**,**d** exhibited more activity than the corresponding 13-*n*-hexyl derivatives **4a**,**c**. Among them, compound **4b** possessed the topmost antitumor activity, achieving a tumor inhibitory rate (TIR) of 59.86% at a dose of 2.5 mg/kg and showed better dose-efficacy relationship. Whereas compound **4a**,**b** and **d** had perspicuously higher toxicity than berberine, palmatine and CP, and produced marked decreases in body weight and even the emergence of the phenomenon of death.

By reducing the dose, the toxic effect of the evaluated compounds can be decreased and their inhibition rate also dropped less. This preliminary *in vivo* test results showed compound **4c** had good TIR and lower toxicity, indicating that it may have potential clinical value for treating cancer. Forthermore, Chen demonstrated the 13-hexyl-berberine gel could achieve effective anti-HSV (herpes simplex virus) concentrations in the dermis and it was safe to use [31]. The deep study on the administration routes and formulations of 13-*n*-alkyl berberine and palmatine analogues will be carried out.

## 3. Experimental

### 3.1. General

All melting points were measured on a Büchi melting point B-540 apparatus (Büchi Labortechnik, Flawil, Switzerland) and were uncorrected. Mass spectra (MS) were taken in ESI model on Agilent 1100 LC-MSD (Agilent, Palo Alto, CA, USA). ^1^H-NMR spectroscopy was performed using a Bruker ARX-300 instrument (Bruker, Fällanden, Switzerland), operating at 300 MHz with TMS as an internal standard. The chemical shifts were reported in ppm (δ) and coupling constants (*J*) values were given in Hertz (Hz). Signal multiplicities were represented by: s (singlet), d (doublet), dd (double doublet), t (triplet), dt (triple triplet), m (multiplet). Elemental analysis was performed by an Elementar Vario EL III instrument (Heraeus GmbH, Hanau, Germany) for C, H, and N and the results were within ±0.5% of the theoretical values. Unless noted otherwise, all solvents and reagents were commercially available and used without further purification.

### 3.2. Chemistry: General Procedure for the Synthesis of Compounds **4a–d**

To a stirred solution of **1a** or **1b** (13.4 mmol) in 5% NaOH solution (100 mL), 5% NaOH (50 mL) solution containing NaBH_4_ (13.4 mmol) was added dropwise. The reaction mixture was stirred at room temperature for 3 h and the precipitated product was filtered, washed with 60% ethanol (20 mL), and then recrystallized from absolute ethanol to provide **2a** or **2b** as a brownlike solid. To a stirred solution of **2a **or **2b** (5.0 mmol) in 80% ethanol (8 mL) and HOAc (2 mL), aldehyde **3a** or **3b** (5.0 mmol) was added. The reaction mixture was heated to 85–95 °C for 5 h. The solvent was removed by evaporation, and the residue was acidiﬁed with 2 mol/L HCl (5 mL), then stirred at room temperature for 1 h. The solid was collected by ﬁltration and then puriﬁed by ﬂash chromatography over silica gel, affording the title compounds **4a**–**d** as yellow powders.

*2,3-Methylenedioxy-9,10-dimethoxy-13-n-hexylprotoberberine chloride *(**4a**). Yield 65%; m.p. 192–193 °C; ^1^H-NMR (CDCl_3_): δ (ppm) 10.66 (s, 1H, Py-H), 7.87–7.94 (m, 2H, Ph-H), 7.12 (s, 1H, Ph-H), 6.90 (s, 1H, Ph-H), 6.11 (s, 2H, -OCH_2_-), 5.28–5.33 (m, 2H, -CH_2_-), 4.33 (s, 3H, -OCH_3_), 4.09 (s, 3H, -OCH_3_), 3.26–3.31 (m, 2H, -CH_2_-), 3.14–3.18 (m, 2H, -CH_2_-), 1.83–1.88 (m, 2H, -CH_2_-), 1.47–1.54 (m, 2H, -CH_2_-), 1.34–1.39 (m, 4H, -CH_2_-), 0.92 (t, 3H, *J *= 7.0 Hz, -CH_3_); ESI-MS *m/z*: 420.1 [M−Cl]^+^; Anal. calcd. for C_26_H_30_ClNO_4_ × 1/2H_2_O: C 67.16, H 6.72, N 3.01; found: C 67.21, H 6.68, N 3.01.

*2,3-Methylenedioxy-9,10-dimethoxy-13-n-octylprotoberberine chloride *(**4b**). Yield 56%; m.p. 188–189 °C; ^1^H-NMR (CDCl_3_): δ (ppm) 10.59 (s, 1H, Py-H), 7.79–7.86 (m, 2H, Ph-H), 7.04 (s, 1H, Ph-H), 6.83 (s, 1H, Ph-H), 6.04 (s, 2H, -OCH_2_-), 5.19–5.24 (m, 2H, -CH_2_-), 4.28 (s, 3H, -OCH_3_), 4.02 (s, 3H, -OCH_3_), 3.17–3.22 (m, 2H, -CH_2_-), 3.07–3.12 (m, 2H, -CH_2_-), 1.78–1.81 (m, 2H, -CH_2_-), 1.43–1.46 (m, 2H, -CH_2_-), 1.11–1.16 (m, 8H, -CH_2_-), 0.81(t, 3H, *J *= 7.0 Hz, -CH_3_); ESI-MS *m/z*: 448.1 [M−Cl]^+^; Anal. calcd. for C_28_H_34_ClNO_4_ × 3/4H_2_O: C 67.59, H 7.19, N 2.82; found: C 67.69, H 7.15, N 2.68. 

*2,3-Dimethoxy-9,10-dimethoxy-13-n-hexylprotoberberine chloride *(**4c**). Yield 64%; m.p. 180–182 °C; ^1^H-NMR (CDCl_3_): δ (ppm) 10.89 (s, 1H, Py-H), 7.89–7.95 (m, 2H, Ph-H), 7.26 (s, 1H, Ph-H), 6.96 (s, 1H, Ph-H), 5.20–5.25 (m, 2H, -CH_2_-), 4.39 (s, 3H, -OCH_3_), 4.16 (s, 3H, -OCH_3_), 4.06 (s, 3H, -OCH_3_), 4.01 (s, 3H, -OCH_3_), 3.28–3.34 (m, 2H, -CH_2_-), 3.18–3.22 (m, 2H, -CH_2_-), 1.91–1.96 (m, 2H, -CH_2_-), 1.54–1.61 (m, 2H, -CH_2_-), 1.42–1.46 (m, 4H, -CH_2_-), 0.96 (s, 3H, *J *= 7.0 Hz, -CH_3_); ESI-MS *m/z*: 436.2 [M-Cl]^+^; Anal. calcd. for C_27_H_34_ClNO_4_ × 1/2H_2_O: C 67.42, H 7.33, N 2.91; found: C 67.71, H 7.45, N 2.99. 

*2,3-Dimethoxy-9,10-dimethoxy-13-n-octylprotoberberine chloride *(**4d**). Yield 62%; m.p. 175–177 °C; ^1^H-NMR (CDCl_3_): δ (ppm) 10.83 (s, 1H, Py-H), 7.85–7.89 (m, 2H, Ph-H), 7.19 (s, 1H, Ph-H), 6.91 (s, 1H, Ph-H), 5.21–5.26 (m, 2H, -CH_2_-), 4.33 (s, 3H, -OCH_3_), 4.07 (s, 3H, -OCH_3_), 3.98 (s, 3H, -OCH_3_), 3.92 (s, 3H, -OCH_3_), 3.25–3.31 (m, 2H, -CH_2_-), 3.18 (s, 2H, -CH_2_-), 1.89–1.91 (m, 2H, -CH_2_-), 1.51–1.54 (m, 2H, -CH_2_-), 1.27–1.32 (m, 8H, -CH_2_-), 0.87 (s, 3H, *J *= 7.0 Hz, -CH_3_); ESI-MS *m/z*: 464.2 [M-Cl]^+^; Anal. calcd. for C_29_H_38_ClNO_4_ × H_2_O: C 67.23, H 7.78, N 2.57; found: C 67.54, H 7.88, N 2.70.

### 3.2. Pharmacology

#### 3.2.1. MTT Assay

The MTT [32] cell proliferation assay was used to test the antitumor activity of berberine, palmatine and compounds **4a**–**d**. The cells were seeded in RPMI-1640 medium (100 μL) in a 96-well plate at a concentration of 5000 cells per well. After culturing for 12 h at 37 °C and with 5% CO_2_, cells were incubated with scalar concentrations of the tested compounds for 24 h. MTT was added to the cultures at a final concentration of 5 μg/mL, and incubated for 4 h. The formazan crystals were formed and dissolved in DMSO (100 μL) each well. The optical density was measured at 570 nm with the reference wavelength 630 nm. All of the compounds were tested thrice independently using the same cell line. IC_50_ (concentration that inhibits 50% of cell growth) was calculated using the Bacus Laboratories Incorporated Slide Scanner (Bliss) software, and the data in this manuscript represent mean of two independent experiments.

#### 3.2.2. Anticancer Activity *in Vivo*

Cyclophosphamide was supplied by Shanghai Hualian Pharmaceutical Co. Ltd. (Shanghai, China); Kunming mice weighing 19–21 g were received from the Shanghai Laboratory Animal Center (Shanghai, China) and kept at five mice/cage at 22–28 °C on a 12 h light/dark cycle with food and water *ad libitum*. All animals were treated in accordance with the guidelines established by the Institutional Animal Care and Use Committee.

The S180 sarcoma tumor cells (supplied by the Pharmacology Laboratory of Shanghai Institute of Pharmaceutical Industry, Shanghai, China) were diluted with 0.9% normal saline solution to 1–2 × 10^7^ cells/mL and transplanted s.c. *via* trocar into the left armpits by using an aseptic manipulation, 0.2 mL/mouse. Cyclophosphamide, berberine, palmatine and compounds **4a**–**d** were rejected by the vein at various doses. Each mouse was weighed three times a week and at the end of the study; tumor was weighted by electron scales and tumor inhibition rate was calculated according to the following formula: 

Tumor inhibition rate = (mean tumor weight of negative group − mean tumor weight of treated group)/mean tumor weight of negative group × 100%.

#### 3.2.3. Statistical Analysis

All data are presented as mean ± standard deviations (S.D.). The confidence limits were set at *p* < 0.05. Statistical significance of the differences between groups was assessed by Student’s *t*-test.

## 4. Conclusions

The present study to investigate the effect of various long carbon-chain subinstituts at the C-13-position of berberines and palmatines on antitumor activity was successfully carried out. The primary screening results indicated that 13-*n*-octyl-palmatine (**4d**) displayed potent cytotoxic activity against seven cancer cells *in vitro*, but 13-*n*-hexyl-berberine (**4c**) exhibited better antitumor activity and less toxic effect *in vivo*. These studies may provide some guidances for the development of natural compounds as anticancer agents with potential clinical value. Further, structure-activity relation studies and mechanistic studies on this new class of berberine and palmatine compounds are currently in progress and will be reported in due course.
